# The influence of illumination and cast shadows on prey detectability by predators

**DOI:** 10.1098/rsos.250719

**Published:** 2025-09-03

**Authors:** Lou Cauchi, Keagan Reynolds, Sami Merilaita, Jennifer Kelley

**Affiliations:** ^1^ LLSHS, University of Sorbonne Paris Nord, Villetaneuse, France; ^2^ School of Biological Sciences, The University of Western Australia, Perth, Australia; ^3^ Department of Biology, University of Turku, Turku, Finland

**Keywords:** background matching, camouflage, countershading, shape perception, visual cues

## Abstract

Many species use camouflage to dissimulate their true form and avoid detection or recognition. In natural habitats, the three-dimensional structure of an organism’s body can present challenges for camouflage, as overhead illumination creates luminance gradients (‘self-shadows’) across the body surface and cast shadows (when light is blocked by the object itself) on the surface behind the object. While self-shadows are known to increase prey detectability to predators, it is unclear whether this is also the case for cast shadows. We used computer-generated prey and live fish as predators (western rainbowfish; *Melanotaenia australis*) to investigate whether the illumination conditions and the presence of cast shadows increase the detectability of prey. In the first experiment, the background contained directional illumination cues, while in the second experiment, targets were presented on a homogeneous grey background. In both experiments, we found that neither the illumination conditions nor the presence of a cast shadow (nor their interaction) increased the probability of detection by predators, despite differences in luminance variation among the different prey stimuli. Our findings suggest that cast shadows do not provide additional contrast to that produced by self-shadows and that cast shadows do not provide depth cues that increase prey detectability by predators.

## 1. Introduction

Many animals use camouflage to dissimulate their true form and avoid being detected or recognized by predators [1]. This adaptation is crucial for survival, as it allows prey to escape predation and predators to ambush their prey [2,3]. In natural habitats, the three-dimensional (3D) structure of an organism’s body can present challenges for camouflage, as lighting conditions create luminance gradients (‘self-shadows’) across the body surface. These self-shadows can potentially increase the detectability of prey due to increased contrast across the body’s surface. For example, Donohue *et al*. [4] used fish as predators to demonstrate that 3D prey can easily be detected by predators, even when their luminance was matched, on average, to that of the background. This finding is supported by theoretical models and empirical studies showing that 3D prey will never be perfectly camouflaged against a uniform background because of the increased contrast created by changes in luminance across the surface of the prey’s body [5–8].

Animals can reduce or remove their self-shadows using countershading colouration, a colour pattern gradient in which the side of the body receiving the strongest illumination (typically the dorsal surface) is darker than the opposite side [9]. This type of colouration is found in many terrestrial and aquatic animals, such as primates [10], artiodactyls [11], sharks [12], fishes [13] and cetaceans [14]. Countershading colouration is thought to enhance camouflage via several (not mutually exclusive) mechanisms, including reducing contrast across the body, increasing background matching and removing 3D shape cues [7,15]. The latter mechanism may occur if luminance gradients provide valuable information on an object’s 3D shape and predators have a search image for 3D objects and their associated visual cues [4,7]. Indeed, animals such as macaques [16], pigeons [17] and cuttlefish [18] are able to recognize and discriminate among 3D shapes using the information from self-shadows, an ability known as ‘shape-from-shading’ [19].

While self-shadows are known to be critically important for camouflage, less is known about the role of other types of shadow, such as cast shadows, which are shadows that are projected onto the substrate behind the object [20,21]. Cast shadows are highly relevant for the perception of spatial information in humans [22]; for example, adults use cast shadows to determine the orientation of an object’s surface and to perceive the 3D shape of objects [23,24]. Additionally, cast shadows provide crucial information for perceiving distances and the movement of objects within 3D space [25]. There is some evidence that cast shadows are important for camouflage; using humans in a visual search task, Adams *et al*. [26] found that camouflaged snakes were detected more rapidly when cast shadows were present than when they were absent, suggesting they provide a depth cue that promotes image segmentation and object recognition. European cuttlefish (*Sepia officinalis*) alter their camouflage patterns in response to objects with strong cast shadows, suggesting that cast shadows play a role in camouflage, but without providing depth cues [27]. With the exception of the abovementioned examples, the role of cast shadows in camouflage has generally received little attention [28].

The production of shadows (both self-shadows and cast shadows) is dependent on the illumination conditions, the illumination angle and the orientation of the object. For example, directional illumination, such as that produced on a sunny day, produces stronger shadows than diffuse illumination when the sky is overcast. In the case of cast shadows, overhead illumination produces smaller shadows that are closer to the object than shadows that are produced at small angles of incidence (i.e. dawn and dusk). The illumination conditions can therefore have an important effect on an animal’s camouflage strategy. For example, bearded dragons, *Pogona vitticeps*, change colour according to the visual background and lighting conditions, illustrating the impact of lighting on an animal’s ability to match its visual background [29]. For animals that cannot change colour, the illumination conditions can determine the effectiveness of camouflage. In the case of countershading colouration, ‘optimal’ camouflage only exists for very specific lighting (i.e. weather) conditions [5] and orientation angles [6,30]. In fact, when countershading colouration is optimal for the illumination conditions (e.g. strong countershading in sunny conditions), detection times are slower than when the prey are incorrectly camouflaged for the conditions or are uniformly coloured [8]. An animal’s activity levels depend on the risk of predation because animals that are poorly camouflaged for the illumination conditions must behave in a risk-averse manner [31]. Indeed, marine isopods that are exposed to diffuse light had higher activity levels, particularly on backgrounds that mismatched their colouration, than those exposed to direct light [31]. Although the effectiveness of camouflage strategies is intricately linked to the illumination conditions, the role of shadows, particularly cast shadows, in prey detectability is generally not well understood.

The aim of this study was to determine the influence of a prey’s cast shadow on predator detection behaviours under two different illumination conditions: diffuse illumination and direct illumination. We used western rainbowfish (*Melanotaenia australis*) as predators, which were trained to detect and approach computer-generated prey. In aquatic environments, the light is generally diffuse, but the production of shadows in shallow, clear water habitats may make it easier for predators to detect their prey [32]. Furthermore, this species of fish has been used in previous studies investigating the influence of shadows on prey detectability [4]. We conducted two experiments in which prey were rendered under diffuse or direct illumination and presented to fish predators with or without a cast shadow. In the first experiment, the visual background contained directional illumination cues (i.e. the background varied in brightness and was brighter in regions closer to the source of illumination), which may provide additional depth information, while in the second experiment, prey were presented on a homogeneous grey background (i.e. no variation in brightness across the background). We predicted that the presence of a cast shadow would facilitate visual detection by predators and that prey rendered under direct illumination would be detected more rapidly (because directional light produces strong shadows with high contrast) than those rendered under diffuse illumination. We also expected prey to be more detectable on homogeneous backgrounds than on backgrounds containing directional illumination cues, because the latter are more visually complex (contrast varies across the visual scene), potentially making the detection task more difficult.

## 2. Material and methods

### 2.1. Study species

We measured the detection behaviour of western rainbowfish, *M. australis* (Castelnau, 1875), which had been trained to approach and ‘attack’ computer-generated prey. This species is a small freshwater fish (total length less than 10 cm) endemic to northern Western Australia. Rainbowfish inhabit a variety of freshwater habitats, including creeks, pools and springs; thus, the natural lighting conditions range from bright, clear waters to turbid pools containing large amounts of algae or tannin [33]. Rainbowfish are omnivorous, opportunistic feeders with a diet that includes aquatic and terrestrial invertebrates, filamentous algae and fish eggs [34]. We chose to work on rainbowfish due to their adaptability to laboratory conditions, their advanced cognitive abilities and their aptitude for learning spatial association tasks [35]. Wild-caught adult fish were housed socially (group size: 4–10 fish) in aquaria measuring 50 × 35 × 35 cm^3^ (*L* × *W* × *H*), maintained at a temperature of 26°C (± 1°C) and subjected to a 12 h light : 12 h dark cycle. In this study, we used the same fish in both experiments: nine males and thirteen females in experiment 1, and seven males and twelve females in experiment 2. This project was approved by the University of Western Australia (UWA), Animal Ethics Committee, under ethics protocol ET000668.

### 2.2. Computer-generated prey

Throughout training and testing, the size and contrast of the prey stimuli were progressively adjusted to facilitate learning and assess generalization. In early training sessions, fish were presented with large, high-contrast stimuli, and stimulus size was reduced either within a single day or across days, depending on the phase. In later stages of training, prey were displayed in more complex visual contexts, using either homogeneous or heterogeneous backgrounds and always with consistent final dimensions of 1.2 × 2.0 cm^2^ during testing. A full summary of stimulus sizes, background type, position and number of presentations for each training and testing phase of both experiments is provided in electronic supplementary material, table S1.

The prey were modelled as an oval shape measuring 1.2 cm in width and 2.0 cm in length, representing a potential food item for the rainbowfish. To train the fish, we used the graphics software Inkscape (inkscape.org) to generate ovals with different shades of grey (greyscale values from 0 to 255, where 0 = black and 255 = white). We first presented black ovals (0) on a homogeneous grey background with a mean value of 128 (i.e. 0.5 in normalized scale). We then presented ovals with greyscale values of 0 (black), 85 (dark grey) or 185 (light grey) on a heterogeneous grey background in experiment 1 (i.e. a structured pattern simulating a naturalistic lighting gradient, with pixel values varying in a way that mimics directional illumination, mean grey value = 128) and on a homogeneous grey background in experiment 2. The heterogeneous background was designed to reproduce more ecologically relevant visual conditions, in which prey are typically viewed against unevenly lit surfaces, including light patches and shadows. We then presented ovals with greyscale values of 0 (black), 85 (dark grey) or 185 (light grey) on a heterogeneous/homogeneous grey background with a mean value of 128. Finally, we presented ovals with a luminance gradient (greyscale range = 46–182) on the same heterogeneous (experiment 1) or homogeneous (experiment 2) backgrounds. Thus, most stimuli were darker than the background, except for one stimulus, which was on average lighter than the background. In experiment 2, ovals were presented on homogeneous grey backgrounds throughout training and testing. For the test trials in both experiments, we generated stimulus prey that were 3D oval objects (ovoids) using the open-source computer animation software Blender (http://www.blender.org). Use of this 3D animation software allowed us to model prey under different types of illumination (direct light or diffuse light), different illumination directions (from the left or the right) and to model prey with and without cast shadows. We chose to use illumination from the left or the right because this is the typical scenario in nature (light is only directly overhead for a short duration). Modelling of cast shadows required a substrate for the shadows to be projected onto, so the 3D modelling procedure also allowed us to position the ovoid above a horizontal substrate.

**Figure 1.**
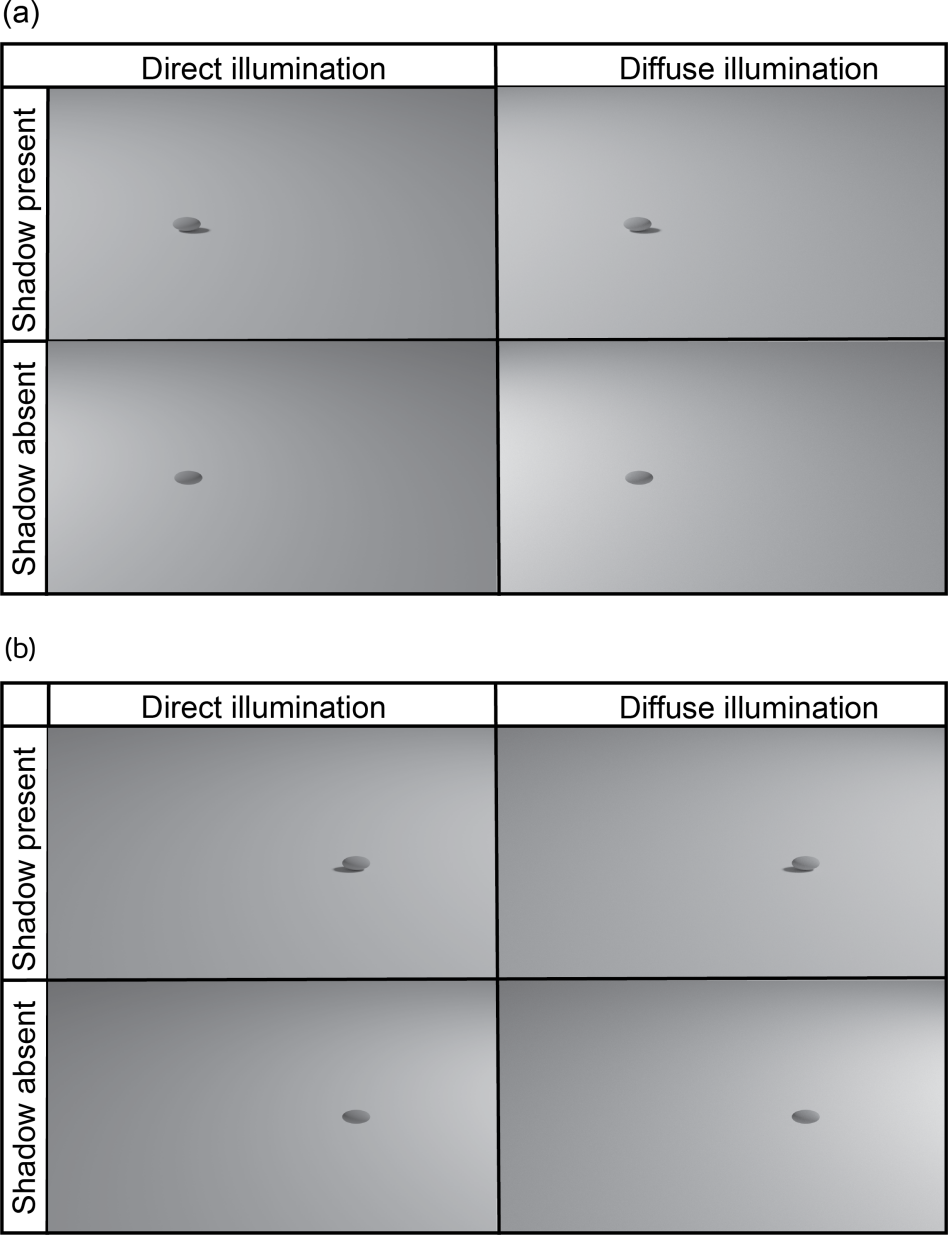
Examples of the prey stimuli that were presented to western rainbowfish on a computer monitor. Prey stimuli were ovoids (1.2 × 2.0 cm^2^) lit by a direct or diffuse source of illumination and rendered using Blender™ 3D modelling software with or without a cast shadow. During day 1 of training, ovoids were presented in the centre of the screen. During subsequent training and testing, ovoids were presented on the left (a) or right (b) of the computer screen, with the illumination coming from the left (a) or the right (b), yielding a total of 16 combinations (not all possible combinations shown).

In Blender, an idealized 3D ovoid (width = 1.2 cm, length = 2.0 cm) was exposed to an overhead light source and positioned 1.3 cm above a horizontal plane (25 × 25 cm^2^). Diffuse lighting was modelled by adding a 7.5 × 7.5 cm^2^ partially transparent plane, angled at 45^o^ and positioned between the ovoid and the light source. Cast shadows were removed by adding transparency and a mix shader to the ovoid’s output material. All images were rendered from a lateral viewing angle with illumination from the left and right for each lighting treatment for prey with and without cast shadows (cycles render engine; *n* = 1000 samples). In the test trials, ovoids were presented on the left or right of the screen and were stationary in all treatments, giving a total of 16 greyscale .TIF images (figure 1). The illumination direction was altered to control for any potential bias that might influence object detection/recognition (e.g. for human vision [36]). An additional set of stimuli was generated for training day 3 using the methods described above, but with illumination angles of 20^o^ and 70^o^. We determined the visual properties of our test stimuli using ImageJ to measure the luminance range (maximum greyscale value − minimum greyscale value) of each test ovoid and its cast shadow (if present). We chose to focus on the luminance range rather than other measures (e.g. average luminance), as this has been shown to represent the largest source of contrast [4].

To generate the backgrounds used during fish training and testing (for experiment 2), we used Matlab to generate images (1920 × 1080 pixels), which were a homogeneous grey (all pixels with a greyscale value of 128). During fish training, ovals were presented on grey or heterogeneous backgrounds (electronic supplementary material, table S1). For training days two and three and testing in experiment 1, the background was the output of the Blender rendering so that the visual background included information about the direction of lighting and shadows. This resulted in backgrounds with varying brightness, where the background was brightest in the region closest to the source of illumination and darkest in regions further away from the light source. In experiment 1, both the prey and the background therefore changed with each presentation. For simplicity, visual scenes are referred to as ‘stimuli’ hereafter. In experiment 2, we followed the same training and testing procedure, but prey ovals were presented on a homogeneous grey background (uniform grey, luminance = 128), so there was no variation in background brightness associated with the illumination source.

### 2.3. Experimental apparatus and procedure

**Figure 2.**
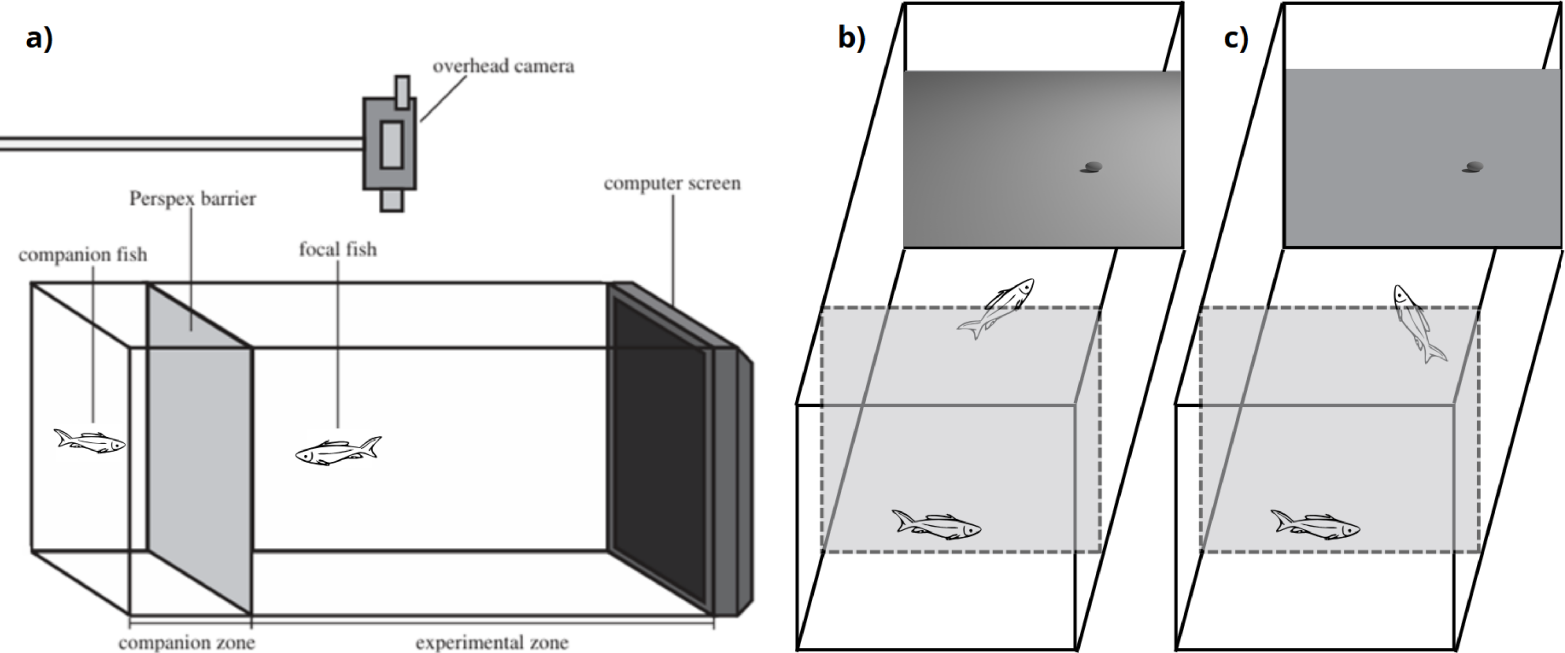
Experimental set-up. (a) Front view of the experimental tank showing the companion zone (left), containing a companion fish, separated from the experimental zone (right) by a transparent Perspex barrier (redrawn from [8]). An overhead camera records the behaviour of the focal fish. (b) Example of the set-up used in experiment 1, where an oval stimulus (1.2 × 2.0 cm^2^ on screen) is presented on a background with directional illumination. In this example, the stimulus is positioned on the right side of the screen, with a shadow, and direct illumination coming from the right. (c) Example of the set-up used in experiment 2, where the stimulus is presented on a homogeneous background. In (b,c), the dashed rectangle indicates the Perspex barrier separating the companion zone from the experimental zone.

All experiments were carried out in the Native Fish Aquarium in the School of Biological Sciences at the UWA. We used a similar experimental set-up (figure 2) to that described in previous visual detection experiments with this species [4]. Training and experiments were done in an 87 l aquarium measuring 76 × 30 × 38 cm^3^ (*L* × *W* × *H*) with opaque walls. An LCD computer monitor (Samsung S24A450BW) replaced one end panel of the aquarium, allowing us to eliminate refraction at the glass–air interface, reducing optical distortion of the computer-generated prey. A transparent silicone tube (diameter = 5 mm) was attached to the screen directly above the locations where prey stimuli appeared to provide a remote food reward during training and experimental trials. Presentation of the stimuli during training and testing was controlled using custom-written code and Psychtoolbox-3 (http://psychtoolbox.org/) for MATLAB.

We placed each focal fish in the aquarium 3 min before the first trial of each training or test session for a period of acclimatization. A ‘companion fish’ was added in a separate compartment to the focal fish, which provided a social stimulus for the focal fish and provided some control over the fish’s location when a stimulus appeared. The Perspex divider separating the focal fish from the companion fish was covered with a one-way mirror window film, preventing the companion fish from seeing the prey. A small lamp was placed above the compartment containing the companion fish to facilitate the one-way mirror. The rest of the experimental tank (including the main compartment containing the focal fish) was lit by the overhead lighting in the observation room. Each stimulus was presented when the focal fish was located in the far section of the aquarium (near the companion fish) and oriented approximately parallel to the dividing wall with the companion compartment. This ensured that fish were approximately the same distance and angle from the monitor when the stimulus appeared. When fish approached within one body length of the monitor in response to the prey stimulus, they were rewarded with food (bloodworm) to maintain motivation during training and testing. The time (between 30 and 120 s) and position of the stimulus (centre position for day 1 of training, presented on the left or right for subsequent training and testing days) between all tests were randomized to avoid the fish anticipating the presentations.

### 2.4. Training and testing of fish

We conducted separate training trials prior to each experiment to train each fish to associate the appearance of prey on the computer screen with a food reward (bloodworm). Fish (*n* = 22 in experiment 1 and *n* = 19 in experiment 2) were trained individually over three consecutive days before the start of the test for each experiment. Training sessions were conducted in the morning or afternoon, and this schedule was maintained during both the 3 day training periods and the experimental tests to avoid any effect of time of day on behaviour. Fish were individually identifiable using sex (males have more pointed dorsal and anal fins), body length and features of their colouration. For the first training day, the fish was exposed to a large (7.1 × 14.4 cm^2^), high-contrast black oval stimulus positioned in the centre of a homogeneous grey background. The size of this stimulus was progressively reduced over the training days until the size was slightly smaller (1.8 × 0.9 cm^2^) than that of the test prey. Starting training with a large stimulus at the centre of the screen increases the chances of the fish developing an association between the prey appearing on the computer screen and a food reward. A custom-written script in MATLAB Psychtoolbox-3 was used to manipulate stimulus size (range: 200.9 × 407.5 pixels (7.1 × 14.4 cm^2^) to 34 × 56.7 pixels (1.2 × 2.0 cm^2^)). Fish were rewarded with bloodworm when they approached within one body length of the screen. During the first training day, we presented each oval stimulus four times, in order from the largest to the smallest. To succeed, fish had to approach at least once for each stimulus size and on all trials with the smallest stimulus. Trials were stopped after a maximum of 25 min if the fish did not succeed, and the fish was removed from the tank. Fish received a reward each time they approached the screen while the stimulus was visible. During other training days, fish had to remain motivated until the last stimulus was presented and maintain a response success rate above 75% to continue. Each training session lasted a maximum of 35 min.

During the second training session, the task became more difficult by introducing a heterogeneous background (for experiment 1) and stimuli that were presented on the right or left side of the screen. These ovals (3.6 × 7.2 cm^2^, 1.8 × 3.6 cm^2^ and 0.9 × 1.8 cm^2^) were different shades of grey to reduce contrast (electronic supplementary material, table S1). The position of each stimulus during each trial for each fish was randomized (determined before each trial) to avoid the fish learning a sequence of events rather than detecting the side on which the stimulus appeared. Fish were only rewarded when they approached within one body length of the screen and entered the correct ‘zone’ (i.e. the area where the stimulus appeared; left or right) on first approach. In the last training session on the third day, 3D stimuli were presented, consisting of a random combination of prey illuminated from different angles (20 and 70° illumination) and sides (illuminated from the left or right). 3D images were introduced with or without shadows and appeared on the left or right side of the screen. This ensured that at the end of training, fish were familiar with ovoids, shadows and different illumination angles, without experiencing the treatment stimuli.

Testing took place on the day following the last training session. Only fish that responded quickly and consistently to all prey stimuli took part in the experiment (*n* = 17/22 in experiment 1 and *n* = 15/19 in experiment 2). On the test day, fish were presented with all 16 stimuli varying in illumination condition (direct or diffuse), lighting direction (left or right), screen position (left or right) and cast shadow (present or absent). In experiment 1, the visual background was the scene in which the ovoids were rendered and contained directional illumination cues (regions of brightness and darkness in the background). In addition, we used four additional control stimuli corresponding to a background without prey (but with directional illumination information as described above) to ensure that the fish approached the screen when they detected prey, not when the background changed. Thus, each fish was exposed to 20 stimuli, presented in a pre-determined and random order.

In the second experiment, rendered objects were presented on a homogeneous grey background; thus, the prey appeared without a change in the background. The second experiment was conducted four months after the first, using the same individual fish in order to investigate the null results of experiment 1 and to specifically assess how changes in background conditions influence fish detection behaviour. By changing the background to a homogeneous one, we aimed to determine whether the fish adapted their responses according to the environmental cues and whether this could impact prey detection. For experiment 1, when switching between stimuli, the background, its associated lighting and the prey item appeared simultaneously. In experiment 2, the background remained unchanged and only the stimulus appeared. In both experiments, each stimulus was presented for a maximum of 20 s or until the fish approached and took its reward, at which point the stimulus was removed. No transition effects (e.g. fade-ins or fade-outs) were used between stimuli. Fish behaviour was recorded using a camcorder (Sony HDR-CX625) placed directly above the aquarium. At the end of the trials, fish body length was determined (standard length (SL) to the nearest mm) by removing each fish from the aquarium and gently placing the body next to a ruler.

### 2.5. Measuring fish behaviour

Video images were captured at 25 frames s^−1^ and analysed using the software BORIS (v. 8.21.8) [37] to measure the following five predator detection behaviours:

(1) *Probability of approach*. The probability of the fish approaching the screen within 20 s of the stimulus appearing. This was treated as a binomial variable, coded as 1 if the fish was close to the screen (i.e. came within less than one body length of the screen) and 0 if it did not.
(2) *Probability of success*. The probability of the fish choosing the correct side of the screen within 20 s of the stimulus appearing. This was treated as a binomial variable, coded as 1 for success and 0 for no success. Choosing the correct side was defined operationally as the fish approaching the screen (i.e. less than one body length from the screen) on the same side where the stimulus appeared (e.g. approaching the right side of the screen if the stimulus was presented on the right).
(3) *Delay of response*. The time between the appearance of the prey and the start of the fish’s body rotation towards the screen.
(4) *Orientation time*. The time required to orient the body towards the prey after the stimulus appeared.
(5) *Approach time*. The time it took the fish to swim within one body length of the computer screen after the orientation time.

We also calculated the angle of the fish’s body when the stimulus appeared (predator starting angle) and at the end of the fish’s rotation (i.e. before the fish started to move towards the screen) using the software ImageJ (v. 1.54 g) [38].

### 2.6. Effect of stimulus presence

During our experiments, we noticed that fish were continually checking the monitor when no prey stimulus was displayed. To confirm that fish had learned the prey detection task, we therefore measured the approach time of fish that approached the monitor when no stimulus was present. This was done by randomly selecting a frame in each video (using a random number generator) and recording the approach time (no stimulus present) closest to that time point. Approach time was measured in the same way as described above for each fish tested in experiment 1. In experiment 2, to ensure that fish were responding to the prey stimulus rather than the screen itself, we waited for each fish to stop checking the monitor before presenting a new stimulus. Prey stimuli were also presented on a homogeneous background so that only the stimulus appeared, without any change in background between trials.

### 2.7. Luminance of stimuli

To confirm that the prey stimuli varied in their visual characteristics, we used the oval selection tool in ImageJ to select all pixels in the ovoid and the polygon tool to create a region of interest around the cast shadow. We then measured the minimum, mean and maximum greyscale values of these areas, representing luminance of stimuli with (ovoid + cast shadow) and without (ovoid selection; self-shadow only) a cast shadow. We then calculated four possible sources of contrast for each of the 16 stimuli presented: maximum contrast, minimum contrast, average contrast and contrast range (brightest − darkest/background). In each case, we used Weber’s contrast (*L*_*s*_ − *L*_*b*_/*L*_*b*_), which calculates the luminance of the stimulus (*L*_*s*_) relative to the luminance of the background (*L*_*b*_).

### 2.8. Data analyses

All statistical analyses were carried out using R software [39] v. 4.2.1. We used generalized linear mixed models (GLMMs) for binomial data to test for the effect of cast shadow (present or absent), illumination conditions (direct or diffuse) and their interaction on the probability of approach and on the probability of success. We used linear mixed-effects models (LMMs) to test for the effect of treatment on orientation time and approach time. Both GLMMs and LMMs used fish identity as a random factor. These models were fitted using maximum likelihood in the lme4 package [40] and were run separately for experiment 1 (background with directional illumination cues) and experiment 2 (homogeneous background). Additional LMMs with Fisher–Pitman permutation tests were done to analyse the effect of delay of response. To improve the distribution of the residuals, approach time and orientation time were transformed using a log_10_ transformation. We tested the fit of each model by comparing it to the null (random effect only) model using log likelihood ratio tests.

We also tested the importance of additional variables such as stimulus order [1–20], which side of the screen the stimulus appeared on (left or right), the body length of the predator (SL), predator starting angle (in degrees) and the sex of the predator. These variables were retained in the final model only if they had a significant effect (*p* < 0.05). We assessed the assumptions of all models by inspecting the distribution of residual values (using Q–Q plots) and examining plots of standardized residuals against fitted values for each model. We investigated differences in the contrast of the stimuli by plotting different sources of contrast of the stimuli under direct and diffuse illumination for targets with and without (self-shadow only present) cast shadows.

## 3. Results

### 3.1. Experiment 1—background with directional illumination cues

**Figure 3.**
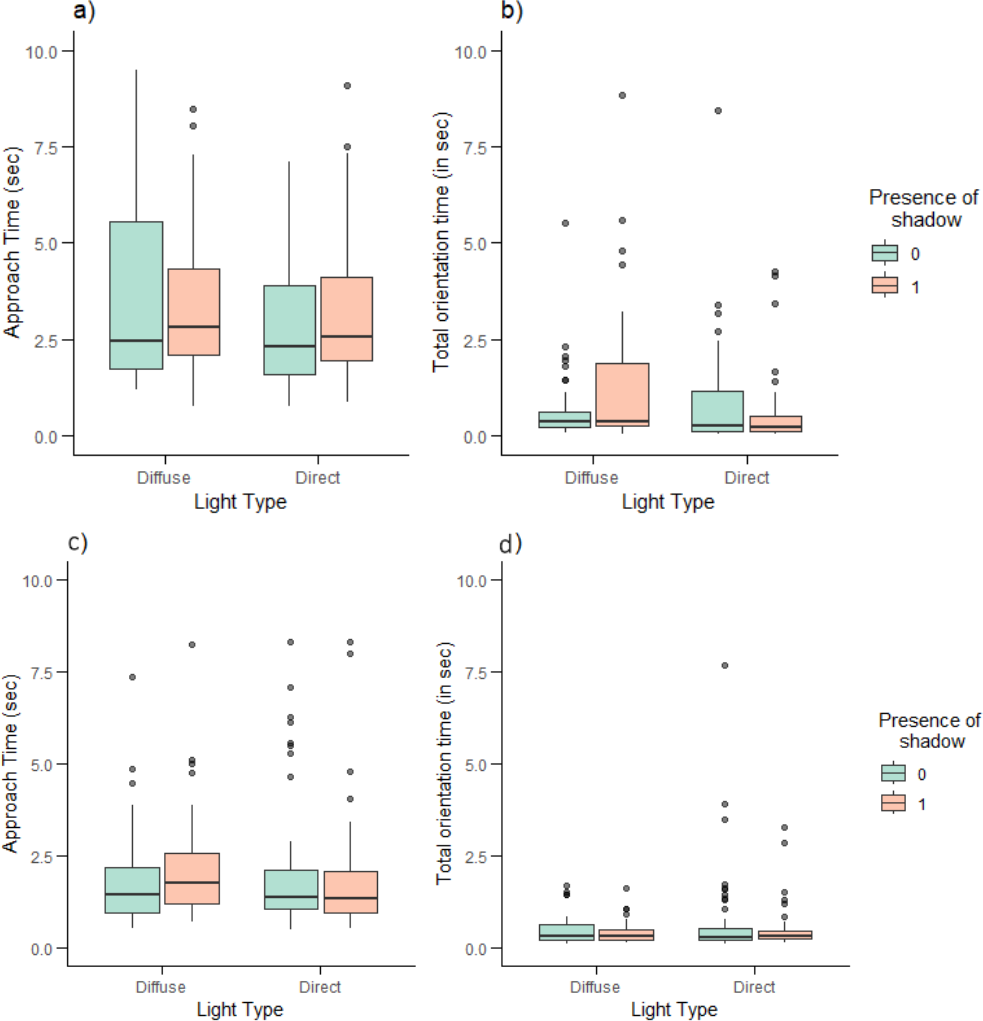
The approach time in seconds (a,c) and the total orientation time in seconds (b,d) depending on the type of light (diffuse: *n* = 80; or direct: *n* = 103), and the presence of cast shadow (0: shadow absent, *n* = 91; 1: shadow present, *n* = 92), for experiment 1 (a,b: background with illumination cues) and experiment 2 (c,d: homogeneous background). Boxes represent the median and interquartile range, and whiskers represent the range of the dataset.

**Figure 4.**
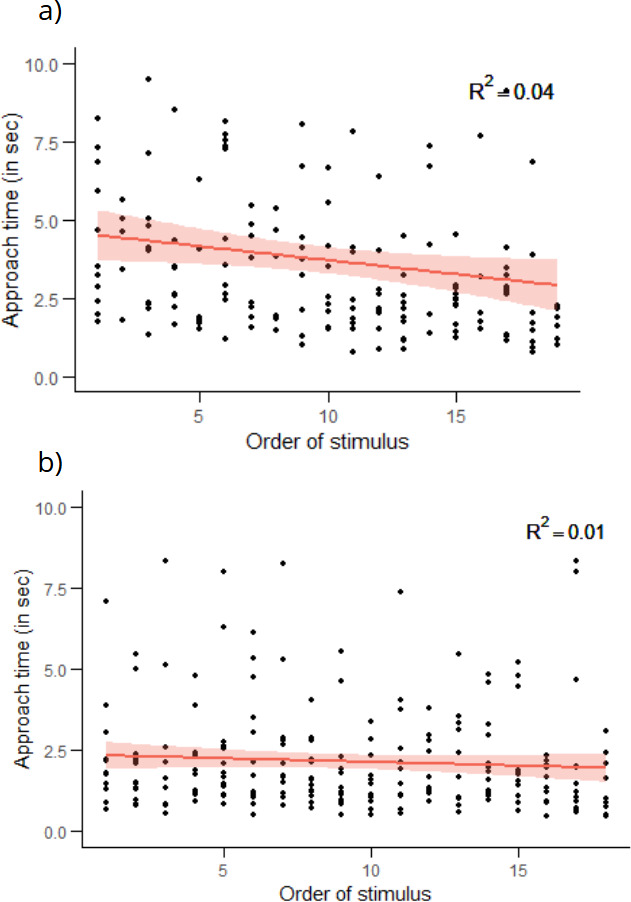
The relationship between approach time in seconds and the order of presentation of stimuli for experiment 1 (a: background with illumination cues; *n* = 18) and experiment 2 (b: homogeneous background; *n* = 15). The regression line is based on parameter estimates from a linear mixed-effects model (see text for details). The area around the curve represents the confidence interval for the prediction of the linear regression.

The interaction between the presence of a cast shadow and the illumination conditions had no significant effect on any of the predator detection behaviours measured (*p* > 0.05; table 1a). There was no effect of the presence of a cast shadow on the probability of predator approach, the probability of success, total orientation time or approach time (table 1a and figure 3a,b). We also found no effect of the illumination conditions on the probability of predator approach, the probability of success, total orientation time and approach time (table 1a and figure 3a,b). There was a significant negative correlation between approach time and the order of presentation of stimuli (LM with permutation test: *β* = −0.015 ± 0.003, *n* = 18; *R*^²^ = 0.04; *p* < 0.001; figure 4a). Additionally, the orientation angle was positively correlated with total orientation time in this experiment (table 1a).

**Table 1.** Results of GLMMs showing the effect of shadow, illumination conditions and body length on the probability of predator approach and the probability of success. Results of LMMs showing the effect of shadow, illumination conditions, body length, orientation angle and stimuli order on the approach time (s) and the total orientation time towards prey (s). Results are shown for experiment 1 (a: background with illumination cues) and experiment 2 (b: homogeneous grey background). The test statistic is *χ*^2^, and the results highlighted in bold are significant.

response variable	fixed effect	slope (± s.e.)	intercept (± s.e.)	d.f.	test statistic	*p*‐value
a						
probability of predator approach	shadow	0.451 (±0.394)	2.171 (±0.433)	1	1.329	0.248
	illumination conditions	−0.187 (±0.389)	2.470 (±0.452)	1	0.232	0.629
	shadow × illumination	−0.403 (±0.791)	2.181 (±0.505)	3	1.813	0.611
	body length	−0.051 (±0.065)	4.889 (±3.531)	4	2.428	0.657
probability of success	shadow	0.148 (±0.284)	0.830 (±0.437)	1	0.267	0.604
	illumination conditions	0.275 (±0.285)	0.771 (±0.436)	1	0.913	0.339
	shadow × illumination	0.297 (±0.571)	0.769 (±0.480)	3	1.45	0.602
	body length	0.022 (±0.075)	−0.400 (±3.994)	4	0.819	0.768
approach time	shadow	0.020 (±0.034)	0.507 (±0.051)	1	0.37	0.542
	illumination conditions	−0.036 (±0.034)	0.536 (±0.052)	1	1.072	0.3
	shadow × illumination	0.115 (±0.069)	0.556 (±0.058)	3	4.264	0.234
	body length	−0.011 (±0.008)	1.172 (±0.475)	1	1.828	0.176
	orientation angle	−0.0001 (±0.000)	0.564 (±0.067)	4	4.321	0.364
	**order**	**−0.015 (±0.003)**	**0.684 (±0.062)**	**1**	**24.874**	**<0.001**
total orientation time	shadow	−0.013 (±0.075)	−0.328 (±0.093)	1	0.02	0.868
	illumination conditions	−0.109 (±0.075)	−0.280 (±0.092)	1	2.127	0.144
	shadow × illumination	−0.149 (±0.151)	−0.313 (±0.106)	3	3.142	0.37
	body length	0.018 (±0.015)	−1.262 (±0.828)	1	1.429	0.231
	**orientation angle**	**0.004 (±0.001)**	**−0.664 (±0.128)**	**4**	**28.928**	**<0.001**
	order	0.001 (±0.007)	−0.327 (±0.122)	1	0	1
b						
probability of predator approach	shadow	−0.863 (±0.555)	4.367 (±1.071)	1	2.546	0.111
	illumination conditions	0.634 (±0.535)	3.529 (±0.998)	1	1.424	0.233
	shadow × illumination	0.421 (±1.120)	4.229 (±1.196)	3	3.715	0.294
	**body length**	**−0.238 (±0.093)**	**16.053 (±5.232)**	**1**	**6.182**	**<0.05**
probability of success	shadow	0.433 (±0.531)	2.101 (±0.531)	1	1.07	0.3
	illumination conditions	−0.313 (±0.426)	2.484 (±0.572)	1	0.548	0.458
	shadow × illumination	−0.277 (±0.862)	2.20 (±0.651)	3	1.553	0.67
	body length	0.047 (±0.079)	−0.238 (±4.110)	4	1.927	0.749
approach time	shadow	0.003 (±0.032)	0.226 (±0.052)	1	0.012	0.911
	illumination conditions	−0.033 (±0.032)	0.246 (±0.053)	1	1.082	0.298
	shadow × illumination	−0.069 (±0.066)	0.224 (±0.060)	3	2.199	0.532
	body length	0.001 (±0.008)	0.159 (±0.467)	1	0.032	0.857
	**orientation angle**	**−0.001 (±0.000)**	**0.389 (±0.084)**	**4**	**11.002**	**<0.05**
	**order**	**−0.007 (±0.003)**	**0.291 (±0.066)**	**1**	**5.328**	**<0.05**
total orientation time	shadow	−0.023 (±0.045)	−0.417 (±0.056)	1	0.266	0.605
	illumination conditions	0.028 (±0.045)	−0.444 (±0.057)	1	0.38	0.537
	shadow × illumination	0.030 (±0.092)	−0.423 (±0.069)	3	0.704	0.872
	body length	0.005 (±0.009)	−0.731 (±0.476)	1	0.531	0.465
	**orientation angle**	**0.003 (±0.001)**	**0.004 (±0.100)**	**4**	**31.113**	**<0.001**
	order	0.003 (±0.093)	−0.453 (±0.080)	1	0	1

### 3.2. Experiment 2—homogeneous background

The interaction between the presence of a cast shadow and the illumination conditions had no significant effect on any of the predator detection behaviours (*p* > 0.05; table 1b).

As in the first experiment, there was no effect of the presence of a cast shadow on the probability of predator approach, the probability of success, total orientation time or approach time (table 1b and figure 3c,d). In addition, the illumination conditions had no significant effect on the probability of success, total orientation time or approach time (table 1b and figure 3c,d). There was a significant effect of body length on the probability of predator approach, whereby larger fish were less likely to approach the screen than smaller fish (table 1b). As in the first experiment, there was a significant negative relationship between the order of presentation of stimuli and approach time (LMM: *χ^2^* = 5.328, *β* = −0.007 ± 0.003, *p* < 0.05; figure 4b). Orientation angle was also positively correlated with total orientation time and with approach time (table 1b).

### 3.3. Effect of prey stimulus presence on approach time

**Figure 5.**
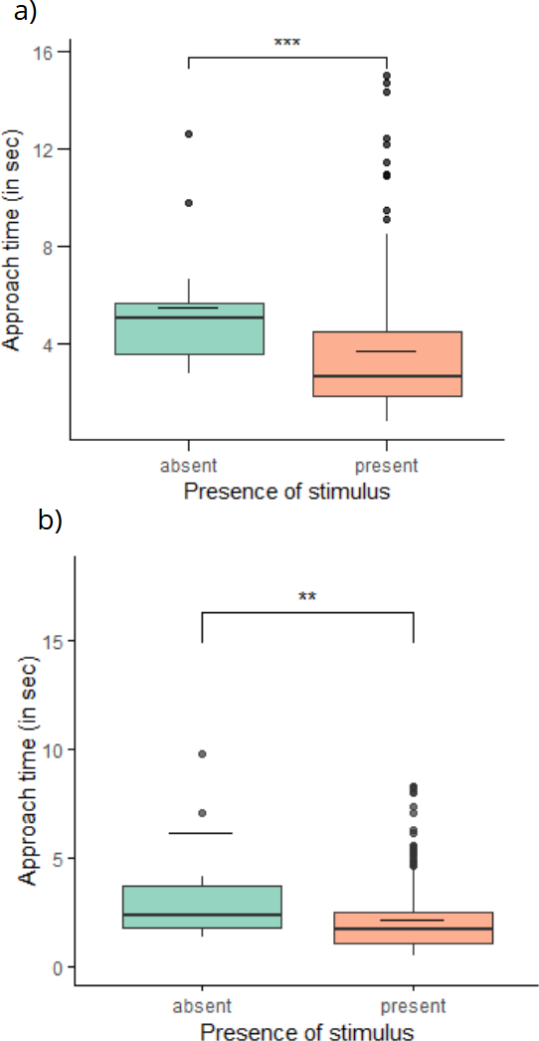
The approach time in seconds according to the presence of the stimulus (present: *n* = 173; absent: *n* = 17) for experiment 1 (a: background with illumination cues) and experiment 2 (b: homogeneous background). Boxes represent the median and interquartile range, and whiskers represent the range of the dataset. ***p < 0.001 from ANOVA main effects, and **p < 0.01, as described in the text.

There was a significant difference in approach time as a function of stimulus presence for both experiment 1, background with directional illumination cues (LMM with exact permutation test, *F*_2,187_ = 11.21, *β* = 0.117 ± 0.035, *p* < 0.001; figure 5a), and experiment 2, homogeneous background (LMM with exact permutation test, *F*_2,223_ = 3.14, *β* = 0.11 ± 0.049, *p* < 0.01; figure 5b). In both cases, the approach time was significantly faster when the stimulus was present compared with trials where it was absent.

### 3.4. Luminance range of stimuli

**Figure 6.**
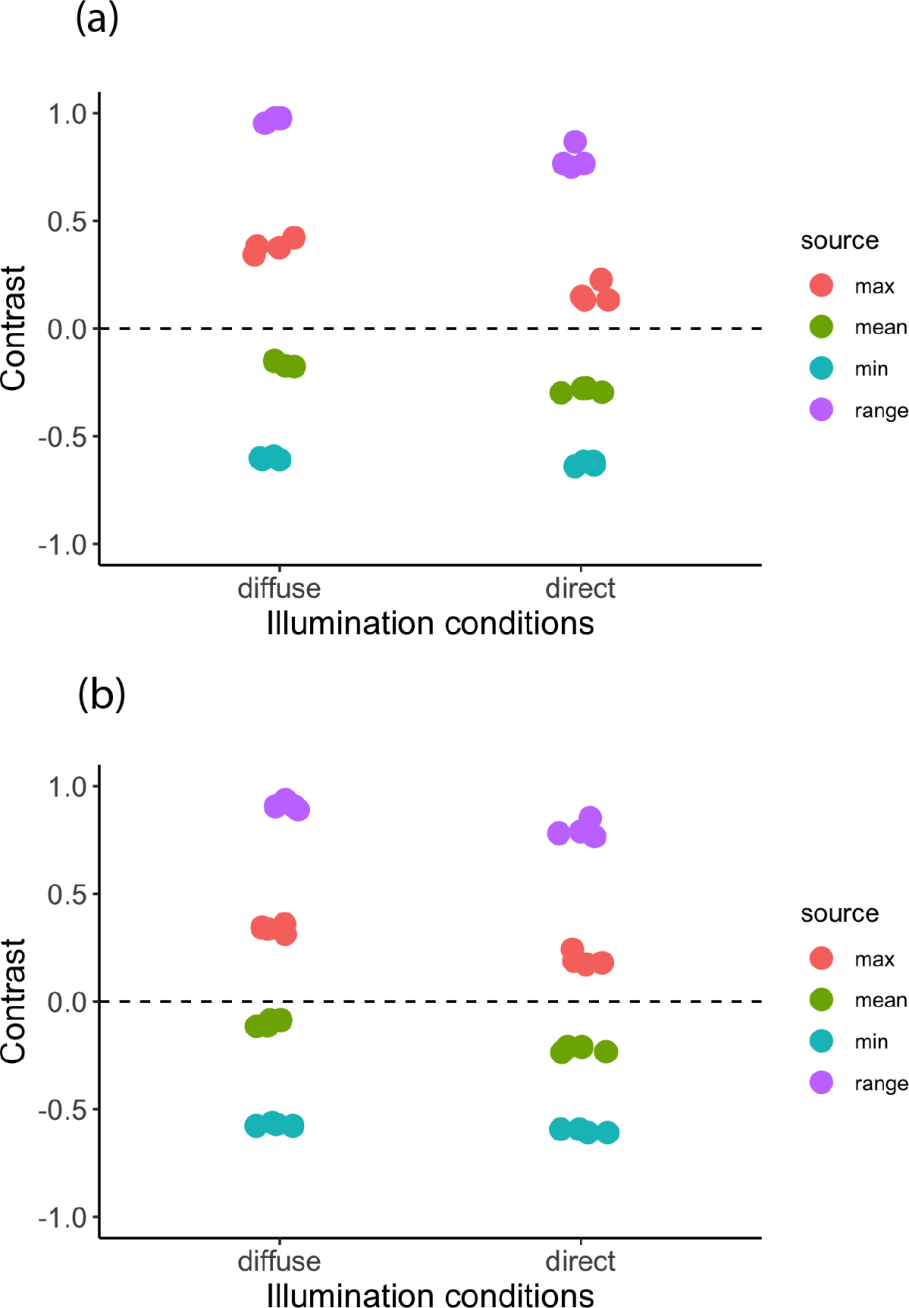
Sources of contrast for the 16 test stimuli for prey rendered under diffuse or direct illumination without a cast shadow (a) or with a cast shadow (b). The sources of contrast were minimum, maximum and mean contrast along with contrast range (maximum − minimum/background). Contrast values above zero are brighter than the background (greyscale value = 128), and contrast values less than zero are darker than the background.

Stimuli had similar contrast values, irrespective of whether they had a cast shadow or not (figure 6a,b). Considering the effect of illumination conditions on contrast, stimuli without cast shadows had slightly higher contrast when rendered under diffuse illumination than under direct illumination (contrast values; diffuse: 0.95−1.03; direct: 0.75−0.87; figure 6a). For stimuli with and without cast shadows, the main source of contrast was the range in contrast caused by the prey’s self-shadow (contrast values: 0.75−1.03), and this was highest where the stimulus was brighter than the background (i.e. positive contrast values).

## 4. Discussion

Cast shadows could potentially provide an important source of information to predators because they can reveal crucial information about an object’s size, shape and location [23–25]. However, our experiments using freshwater fish as predators do not support this idea; irrespective of the illumination conditions (direct or diffuse lighting) and background (directional illumination information present or not), the presence of a cast shadow did not increase the probability or speed of detection. This suggests that any increased contrast caused by cast shadows does not provide an additional source of risk, on top of that produced by the prey’s self-shadows [4]. Our findings also suggest that cast shadows do not provide any additional perceptual cues (e.g. revealing the prey’s shape and location) that increase the probability or speed of detection by predators.

Cast shadows could increase prey detectability by increasing the apparent size of the prey, particularly since the cast shadows were attached to the prey and would therefore increase the overall area of the visual stimulus. Prey that are apparently larger (i.e. due to their cast shadow) are expected to be more detectable than smaller prey, but our study found no evidence for this. In the literature on human vision, cast shadows are known to provide important perceptual information, such as the shape of the object as well as an object’s distance and motion [41]. For example, when asked to judge the distance of two rods presented with and without cast shadows, participants were more likely to be incorrect when observing rods without cast shadows than with cast shadows [42]. Cast shadows can also provide important information about the spatial organization of the scene, such as the distance of the object from the surface that the shadow is projected onto (objects closer to the substrate have closer cast shadows) [41]. For example, experiments with adults and young children presented with drawings of ellipses depicted in 3D scenes found that their size and height estimates were more accurate when the ellipses were depicted with cast shadows present than without [43]. In our experiment, the ovoid prey was positioned just above the horizontal substrate; thus, all cast shadows were the same size and shape (they only differed in direction—illumination from the left or the right), but they differed in presence (present/absent).

One explanation for our findings is that the cast shadows in the direct and diffuse illumination treatments contained similar changes in contrast across the surface and therefore did not result in a change in predator detection behaviours. Additionally, inconsistencies between the actual lighting conditions in the experiment (illumination from approximately overhead) and the apparent lighting conditions (ovoids lit from the left or right) could mean that any depth information available in the cast shadows is redundant. This idea has been investigated in humans, showing that reaction times in visual search tasks are faster when searching for cast shadows from upright objects rather than those of inverted images because the shadows are inconsistent with the typical direction of illumination and may therefore be discounted [44] or processed via different mechanisms [45]. There were also inconsistencies in the apparent location of the horizontal plane that the cast shadows were projected onto; the ovoids were modelled in a position just above the plane, but were presented in the water column at the typical depth the fish swim in the tank. As these discrepancies could also affect visual detection/recognition, future experiments could ensure that the location of the tank’s substrate is consistent with the plane used to model the cast shadows. Although the fish had some experience with cast shadows during day 3 of training, additional experience with objects presented on planes under directional lighting may allow a greater opportunity to learn the visual features of 3D scenes.

It could also be that our protocol, where the same object is presented with or without a shadow, is primarily measuring visual detection behaviours rather than the processes associated with the task of object recognition. Another possible explanation for our results is that the detection task may not have been difficult enough for the cast shadow to provide useful visual information to the predator. If prey detection was relatively easy because of the high contrast between prey and background or the absence of distracting elements, the additional depth cues provided by the shadow might have been redundant. In more complex visual environments, where prey blend more effectively with the background or where multiple visual elements compete for attention [46], cast shadows may play a more important role in facilitating detection [26]. Future studies could manipulate background complexity or contrast to determine whether the role of cast shadows in prey detection is more pronounced under more visually challenging conditions.

When we investigated the contrast of our prey stimuli, we found that stimuli in the different treatments (light conditions: direct or diffuse; cast shadow present or absent) had similar values. While the different sources of contrast were highly variable (e.g. mean contrast: 0.1−0.30; contrast range: 0.75−1.03), previous work on the detection behaviour of this species has found that contrast range (i.e. internal contrast caused by the body’s self-shadow) is the most important predictor of detection probability [4]. In support of this, we found that the greatest source of contrast in our stimuli was due to the ovoid’s self-shadows rather than its cast shadows. This leads us to suggest that under our experimental conditions, self-shadows, rather than cast shadows, are the main source of contrast that determines prey detectability. The highest contrast values were associated with regions of the prey that were brighter than the background, suggesting that increased luminance from light reflected off the prey’s dorsal surface may be more important than decreased luminance from the body’s self-shadows. This is supported by countershading theory; modelling the optimal countershading patterning for 3D prey under naturalist illumination conditions reveals that prey should have the darkest colouration on their uppermost surface, but this depends on prey orientation, the lighting conditions and the angle of the sun [6–8,47].

We also found no effect of the illumination conditions (direct or diffuse illumination) on any of the prey detection behaviours measured. This could be because our modelling processes cannot fully replicate natural illumination conditions; indeed, to remove the cast shadow from our stimuli, we had to change the ovoid’s output material, which is not ideal. This may explain why the illumination conditions did not make much difference to the appearance or contrast measures of our test stimuli. However, it is known that direct and diffuse illumination can cause very different self-shadows on an ovoid (strong self-shadows are produced under direct or sunny, illumination) when using more naturalistic modelling software, such as Radiance [6,7]. Use of such software is likely to have made our stimuli more naturalistic and more distinct in terms of contrast. However, previous work, which has investigated both the effects of illumination intensity and the angle of illumination, has found that the latter is the most important in determining the distribution of natural illumination and hence an object’s shadows [7]. Altering the angle of illumination causes differences in the size and location of the cast shadow; this may have a greater effect on detectability than cast shadow presence/absence and could be explored in future research.

Our findings were the same, irrespective of whether the background contained directional illumination cues (experiment 1) or not (experiment 2). The presence of directional illumination cues in the background might be expected to either reinforce the shadow information available to increase detection or could provide a more complex visual background, increasing detection times. Experiments with humans have examined the effects of cast shadows that are inconsistent with the illumination direction or inconsistent with the shape of the object, finding that the reaction times of participants were fastest when both sources of information were consistent and correct and slowest when both sources of information were in conflict [48]. Although these findings suggest that cast shadows and their associated information are important for object recognition, it is not clear whether this is because shadows add additional information that enhances information processing or because inconsistent information from shadows is distracting and slows the speed of responses [48,49]. Adams *et al.* [26] investigated the role of directional illumination in the ability of human participants to detect images of snakes with camouflage patterns (enhanced edges, which provide false depth cues). They found that snakes were detected more easily under directional illumination than under ambient lighting conditions, which they suggested was due to the cast shadows providing depth information that may promote image segmentation and target recognition. However, they also note that in some situations, cast shadows (from the target and from objects in the background) could increase the visual complexity of the scene, which would make target detection more difficult [26].

During experiment 1, we noted that fish were constantly checking the screen when no stimulus was present. This made it difficult for us to distinguish between when fish were responding to a stimulus and when they were checking the screen. In experiment 1, it was possible that fish were responding to a change in the entire visual scene (the background and the stimulus appeared simultaneously) rather than the appearance of the visual stimulus *per se*. For this reason, we repeated experiment 1, being more stringent with our training, by waiting for fish to stop checking the screen before presenting a stimulus and by repeating the experiment by presenting prey on a homogeneous background (i.e. only the stimulus appeared). We also analysed the behaviour of fish when they checked the screen compared to when they responded to a stimulus. We found that in both experiments, fish approached the screen more rapidly when a stimulus was present than when the stimulus was absent. This suggests that the fish have learned to respond to the presence of the stimulus, which confirms their ability to detect the visual cues presented during these experiments.

Collectively, the two experiments and our additional analysis of fish behaviour give us confidence in our null results. In experiment 1, we found that the approach time of fish decreased over successive trials, indicating that fish tended to approach the screen faster as the experiment progressed. Similarly, in experiment 2, the approach time also decreased over successive trials. Although the correlation between trial number and approach time was significant, a simple correlation does not take account of the random effects, which mask the effect of variability among individuals. However, in our LMMs, the effect of stimulus order is estimated while taking account of this inter-individual variability. This suggests that, rather than becoming satiated and losing motivation, fish may have become more familiar with the task or more engaged as testing proceeded.

In summary, this study did not find evidence to support the hypothesis that cast shadows facilitate the detection and recognition of prey by fish predators. The results indicate that neither direct nor diffuse light, nor the presence of a shadow cast by the prey, increases the probability of detection by predators. We suggest that in our experimental set-up, cast shadows do not provide any additional information beyond that provided by self-shadows, which are known to be an important determinant of prey detectability.

## Data Availability

All the data and code needed to reproduce the analyses, along with an example video of fish detection behaviours, are provided on a public online repository [50]. Electronic supplementary material is available online [51].
